# Association Between Housing Affordability and Severe Maternal Morbidity

**DOI:** 10.1001/jamanetworkopen.2022.43225

**Published:** 2022-11-22

**Authors:** Felix M. Muchomba, Julien Teitler, Nancy E. Reichman

**Affiliations:** 1 School of Social Work, Rutgers, The State University of New Jersey, New Brunswick, New Jersey; 2 School of Social Work, Columbia University, New York, New York; 3 Department of Pediatrics, Robert Wood Johnson Medical School, New Brunswick, New Jersey

## Abstract

**Importance:**

The number of people living in unaffordable housing (relative to income) is projected to continue increasing as housing cost inflation outpaces incomes in the US. Although reproductive-aged women have disproportionately high housing costs, particularly around the time of childbirth, data on associations between housing costs and maternal health and the role of publicly supported affordable housing programs in mitigating those associations are lacking.

**Objective:**

To estimate associations between area-level rental housing costs and severe maternal morbidity (SMM) and assess the potential mitigating role of publicly supported affordable housing.

**Design, Setting, and Participants:**

This cross-sectional study linked New Jersey birth files from January 1, 2008, to December 31, 2018, to maternal hospital discharge records and municipal-level housing and demographic data from the state of New Jersey and the US Census Bureau. Data were analyzed from January to September 2022. The birth files contained records for all births in New Jersey, and the hospital discharge records contained information from all inpatient hospitalizations over the study period. A total of 1 004 000 birth records were matched to maternal discharge records and municipal-level data.

**Exposures:**

Municipal-level rental costs relative to income (housing cost burden), availability of publicly supported affordable housing, and housing subsidy per person with an income lower than the federal poverty level.

**Main Outcomes and Measures:**

Severe maternal morbidity was identified using diagnosis and procedure codes developed by the US Centers for Disease Control and Prevention to measure SMM.

**Results:**

Of 1 004 000 mothers (mean [SD] age at birth, 29.8 [5.9] years; 44.7% White), 20 022 (2.0%) experienced SMM. Higher municipal rental housing costs were associated with greater odds of SMM (odds ratio [OR], 1.27; 95% CI, 1.01-1.60), particularly among mothers with less than a high school education (OR, 1.81; 95% CI, 1.06-3.10), and the positive associations decreased at higher levels of affordable housing availability. Among mothers with less than a high school education, the risk of SMM was 8.0% lower (risk ratio, 0.92; 95% CI, 0.85-1.00) for each additional $1000 annual municipal-level housing subsidy per person with an income lower than poverty level after controlling for rental costs and other characteristics, which translated to a 20.7% lower educational disparity in SMM.

**Conclusions and Relevance:**

In this cross-sectional study, living in a municipality with higher rental housing costs was associated with higher odds of SMM, except when there was high availability of publicly supported affordable housing. These results suggest that greater availability of publicly supported affordable housing has the potential to mitigate the association between rental housing costs and SMM and reduce socioeconomic disparities in SMM.

## Key Points

**Question:**

Are area-level rental housing costs associated with severe maternal morbidity (SMM), and does the availability of publicly supported affordable housing attenuate the associations?

**Findings:**

In this cross-sectional study that included 1 004 000 women with birth records in New Jersey, higher municipal rental housing costs were significantly associated with greater odds of SMM. The availability of publicly supported affordable housing appeared to attenuate the associations.

**Meaning:**

These findings suggest that greater availability of publicly supported affordable housing has the potential to mitigate the association between rental housing costs and SMM and reduce socioeconomic disparities in SMM.

## Teaser

This cross-sectional study examines the associations between area-level rental housing costs and severe maternal morbidity and assesses the potential mitigating role of publicly supported affordable housing among mothers in New Jersey.

## Introduction

In 2019, median housing costs grew faster than median household incomes for the eighth year in a row, contributing to an ongoing housing affordability crisis in the US.^1^ Nationwide, 37.1 million households are considered housing cost burdened, meaning that they spend more than 30% of their income on housing.^1^ Women, particularly those of reproductive age with low incomes or educational attainment, have disproportionately high rates of housing cost burden.^1,2,3^ Pregnancy and childbirth are associated with declines in income at a time when housing needs have increased, further exacerbating housing cost burden.^4,5^ In 2019, 57% of women with less than a high school education who gave birth in the past year were burdened by housing costs; this figure is 43% higher than that of women with similar educational attainment who did not give birth.^3^

Housing cost burden is associated with hypertension, arthritis, emergency department use, and poor self-rated health among adults.^6,7,8,9^ Associations between housing cost burden and suboptimal self-rated health are as substantial as (or more substantial than) those between physical housing characteristics (eg, peeling paint or pest infestation) and self-rated health.^7^ Although findings on the association between housing affordability and maternal-reported children’s health have been inconsistent,^10,11^ housing cost burden has been associated with low weight for age (suggesting undernutrition), particularly among children in families with low income.^12^

Although reproductive-aged women have a disproportionately high rate of housing cost burden that increases around the time of childbirth, to our knowledge, the association between housing costs and maternal health has not been previously examined.^13^ This issue represents an important knowledge gap given that 3.7 million births occur each year in the US, with wide socioeconomic and geographic disparities in maternal and infant health.^14,15^ In addition, the US has one of the highest rates of maternal mortality among high-income nations and high rates of severe maternal morbidity (SMM),^16,17^ defined by the Centers for Disease Control and Prevention (CDC) as unintended outcomes of labor and delivery that result in substantial short- or long-term consequences for a woman’s health.^18^

Lack of affordable housing could adversely affect reproductive health through several channels. First, it can lead to crowding, housing instability, or homelessness, which can adversely affect health (eg, through reduced access to reproductive health screenings and timely prenatal care).^19,20,21,22,23^ Second, financial pressure to make timely housing payments can exert a psychological toll,^13,19^ and the threat of losing housing may increase during pregnancy due to perinatal depression or fear of losing custody of a child.^24^ Third, households burdened by housing costs have fewer resources available to spend on health care and nutrition.^1,8,10^ Fourth, the higher rates of household instability in neighborhoods with high housing cost burden may reduce social capital and social support, which are associated with health.^22,23,25^

The US federal government spends more than $40 billion each year on rental assistance programs that states, local governments, and other federal housing programs supplement to varying degrees.^26^ However, affordable housing programs are controversial, with opponents asserting that the programs harm families and communities.^27,28^ To our knowledge, the role of publicly supported affordable housing programs in mitigating the adverse health effects of housing cost burden has not previously been investigated and represents a second important knowledge gap.

This cross-sectional study addressed these 2 key knowledge gaps by linking individual-level birth records from the state of New Jersey to hospital discharge records and municipal-level data on rental housing costs and public housing support programs, with the aim of estimating associations between area-level rental housing costs and maternal morbidity and assessing the extent to which the availability of publicly supported affordable housing may attenuate those associations. We focused on SMM,^16^ a major factor associated with maternal mortality (the worst maternal health outcome) that has similar underlying factors but occurs 70 times more frequently and allows for more robust conclusions.^16^ Severe maternal morbidity also can adversely affect maternal health trajectories and disrupt mother-infant bonding, which can compromise children’s social and emotional development.^29^ The state of New Jersey has the fourth highest maternal mortality rate and one of the highest SMM rates in the nation^15^ as well as substantial variation in public housing policies across municipalities and time as a result of state reforms implemented in 1985 and 2008 (eAppendix 1 in the Supplement). We focused on rental costs, which are more salient than home prices for populations with low income.^1^ We hypothesized that living in areas with less affordable rental housing would be associated with higher odds of SMM, particularly for those with low socioeconomic status, and that greater availability of publicly supported housing would attenuate the associations.

## Methods

This cross-sectional study was approved by the institutional review boards of Rowan University (the institutional review board of record for the New Jersey Department of Health) and Rutgers, The State University of New Jersey. Both institutions approved a waiver of informed consent under the Common Rule (45 CFR §46.116 [f]). The study followed the Strengthening the Reporting of Observational Studies in Epidemiology (STROBE) reporting guideline for cross-sectional studies.

### Data Sources

We obtained individual-level birth records for all births in the state of New Jersey from January 1, 2008, to December 31, 2018. Based on previous research,^14^ we linked these birth records to maternal hospital discharge records from any hospitalizations up to 42 days postpartum. We used probabilistic matching to link records using mother’s name, birth date, address, and hospitalization dates and successfully matched 94% of birth records to at least 1 maternal discharge record. We limited our analysis to births in New Jersey among mothers who resided in New Jersey and used a single birth record for each delivery regardless of plurality. Data were analyzed from January to September 2022.

The residential addresses in the birth records were geocoded, allowing us to link each record to municipal-level characteristics. We used data from the US Census Bureau’s American Community Survey 5-year estimates, which included municipal-level measures of the proportion of renters spending more than 30% of their household income on rent, the proportion of renters spending more than 50% of their household income on rent, median rental cost, poverty rates, and population size. We also collected information on publicly supported affordable housing units from the New Jersey Department of Consumer Affairs,^30^ which included detailed information on the number of housing units and the eligible population (eg, general population, older adults, and individuals with disabilities) by municipality (defined as self-governing administrative divisions incorporated under state law^31^) for each affordable housing program in 2010 and 2015; we used this information to linearly interpolate estimates for the remaining years. The various public programs that provided affordable housing units in the state are shown in eTable 1 in the Supplement.

### Measures

The outcome was whether the mother experienced SMM. The hospital discharge records included diagnosis and procedure codes from the *International Classification of Diseases, Ninth Revision, Clinical Modification* (*ICD-9-CM*) for the years before 2016 or the *Classification of Diseases, Tenth Revision, Clinical Modification* (*ICD-10-CM*) for 2016 and later. We used *ICD-9-CM* or *ICD-10-CM* codes to identify mothers with SMM based on the revised CDC SMM criteria, which include 16 codes for possible life-threatening diagnoses and 5 codes for life-saving procedures (eTable 2 in the Supplement).^16^

Measures of rental costs relative to income (vs absolute rental costs) capture the general level of affordability in rental markets. The primary indicator of municipal-level rental burden was the proportion of renter households spending more than 30% of income on rent based on the US Department of Housing and Urban Development standard for housing cost burden.^32^ Alternative indicators were the proportion of renter households with severe housing cost burden (defined by the Department of Housing and Urban Development as spending >50% of household income on rent^32^) and the ratio of median rental cost to household income (based on previous research^33^).

Our main measure of the availability of affordable publicly supported housing was the ratio of the number of publicly supported affordable housing units (ie, those that were potentially available to anyone who met income requirements, without age or disability restrictions) in the municipality to the number of municipal residents with household income lower than the federal poverty level. In some models, we considered the number of publicly supported affordable housing units that were designated specifically for older adults and calculated estimates per older adult with income lower than the poverty level, which allowed us to assess the validity of our analytical approach.

We also constructed a measure of the implied value of publicly supported affordable housing by assigning, for each affordable housing unit in the municipality, the difference between median annual rental cost in the municipality and the maximum annual rental cost that would be affordable (ie, ≤30% of income) for a household eligible for affordable housing. Because state law required that the mean rental cost for each development be affordable for households earning 52% or less of median income,^30^ the implied value of each housing unit was calculated as median rental cost minus the product of 30%, 52%, and median income. We then computed the total housing subsidy (total implied value) per person with an income lower than the poverty level in each municipality.

Educational attainment (less than high school, high school, some college, or college or higher), obtained from the birth records, was used as an indicator of socioeconomic status. Other individual-level controls, also obtained from the birth records, were self-reported race and ethnicity (non-Hispanic Black, non-Hispanic White, non-Hispanic Asian, Hispanic, or non-Hispanic other or multiple races), maternal age (<20 years, 20-34 years, or ≥35 years), parity (1 birth, 2 births, or ≥3 births), and year of delivery. Municipal-level controls (from the American Community Survey) were median gross rental cost, percentage of residents with income lower than the poverty level, and population size.

### Statistical Analysis

We used a choropleth map to visually assess the extent to which there was geographic clustering in municipal-level housing cost burden in the state. To investigate associations between municipal-level housing cost burden and SMM, we estimated multilevel logistic regression models with a municipality random intercept to account for clustering of observations within municipalities. First, we estimated associations between the proportion of renters in the municipality who were burdened by housing costs and SMM, controlling for individual-level factors (model 1). Second, we included municipal-level factors that may have been associated with both housing cost burden and SMM (model 2). Third, we added an interaction between municipal-level housing cost burden and maternal educational attainment (model 3). Models were estimated using each of the 3 measures of the municipal level housing cost burden. We used complete-case analysis because of low rates of missing data (<1%). Our procedure for testing for spatial autocorrelation in residuals suggested that autocorrelation was not a concern (eAppendix 2 in the Supplement).

Next, to examine whether availability of publicly supported housing modified the associations between municipal-level housing cost burden (using our main measure, the proportion of renter households spending >30% of income on rent) and SMM, we included interactions between municipal-level housing cost burden, availability of publicly supported housing, and maternal educational attainment. We also estimated models using the measure of (implied) housing subsidy per person with an income lower than the poverty level instead of the availability of publicly supported housing, plus an interaction between the implied housing subsidy variable and maternal educational attainment. Housing subsidies decrease housing costs for recipients and thus benefit families that receive them, but they are available to so few families that the subsidies could only minimally reduce housing cost burden at the municipal level. The municipalities offering the highest subsidies (top 1% by number of publicly supported housing units per capita) provided housing for only 7% of their populations.^30^

To test the validity of the analytical approach, we repeated the analysis by substituting availability of publicly supported affordable housing specifically for older adults in place of availability for the general population. If affordable housing for older adults was associated with SMM, it would suggest that our main estimates reflected unmeasured characteristics of municipalities that were associated with the availability of affordable housing. The analysis was conducted using Stata software, MP version 16 (StataCorp LLC). Statistical significance was set at *P* < .05, and all tests were 2-sided.

## Results

The sample included 1 004 000 mothers (mean [SD] age at birth, 29.8 [5.9] years) who delivered in 562 municipalities; 108 781 women (10.8%) were Asian, 148 275 (14.8%) were Black, 281 429 (28.0%) were Hispanic, 448 603 (44.7%) were White, and 16 912 (1.7%) were of other or multiple races (Table 1). A total of 20 022 mothers (2.0%) experienced SMM. Mothers lived in municipalities in which a mean (SD) of 50.4% (10.1%) of residents were rent burdened and a mean (SD) of 11 (20) publicly supported affordable housing units per 100 persons with income lower than the poverty level were available, suggesting a large unmet need for affordable housing. The mean (SD) estimated value of housing subsidy per affordable housing unit was $3768 ($3258) per year (or $314 [$276] per month), and municipalities provided a mean (SD) annual housing subsidy of only $423 ($810) per person with an income lower than the poverty level.

**Table 1.** Characteristics of the Sample^a^

**Characteristic**	**Participants, No. (%) (N = 1 004 000)**
**Individual level**	
Experienced SMM	20 022 (2.0)
Maternal age at birth, y	
Mean (SD)	29.8 (5.9)
Age group	
<20	45 690 (4.6)
20-34	739 230 (73.6)
≥35	219 080 (21.8)
Race and ethnicity	
Hispanic	281 429 (28.0)
Non-Hispanic	
Asian	108 781 (10.8)
Black	148 275 (14.8)
White	448 603 (44.7)
Other or multiple races^b^	16 912 (1.7)
Maternal educational attainment at birth	
Less than high school	120 034 (12.0)
High school	264 094 (26.3)
Some college	203 753 (20.3)
College or higher	416 119 (41.4)
Parity	
1	398 702 (39.7)
2	339 403 (33.8)
≥3	265 895 (26.5)
**Municipality level, mean (SD)^c^**	
Total population	62 450 (69 570)
Percentage with housing cost burden^d^	50.4 (10.1)
Percentage with severe housing cost burden^e^	26.7 (8.2)
Percentage of median rent to income ratio	32.7 (5.9)
Median monthly gross rent, $	1225 (260)
Publicly supported affordable general housing units/person with income lower than poverty level^f^	0.11 (0.20)
Annual housing subsidy, $	
Per housing unit	3768 (3258)
Per person with income lower than poverty level	423 (810)
Publicly supported affordable housing units for older adults/older adult with income lower than poverty level	0.82 (1.43)
Percentage of residents with income lower than poverty level	12.6 (9.6)

Abbreviation: SMM, severe maternal morbidity.

^a^ Data are from linked New Jersey birth and maternal hospital discharge records for 2008-2018, 5-year estimates of the American Community Survey and the New Jersey Department of Community Affairs.

^b^ Other race included individuals who identified as American Indian or Alaska Native; Guamanian or Chamorro; Native Hawaiian, Samoan, or other Pacific Islander, or any other racial group that was not Asian, Black, or White.

^c^ Municipal-level characteristics were weighted by number of births to mothers in each of the 562 municipalities with a birth during the study period.

^d^ Defined as spending more than 30% of income on rent.

^e^ Defined as spending more than 50% of income on rent.

^f^ General housing units are open to anyone who meets the income requirements, without age or disability restrictions.

**Figure 1.**
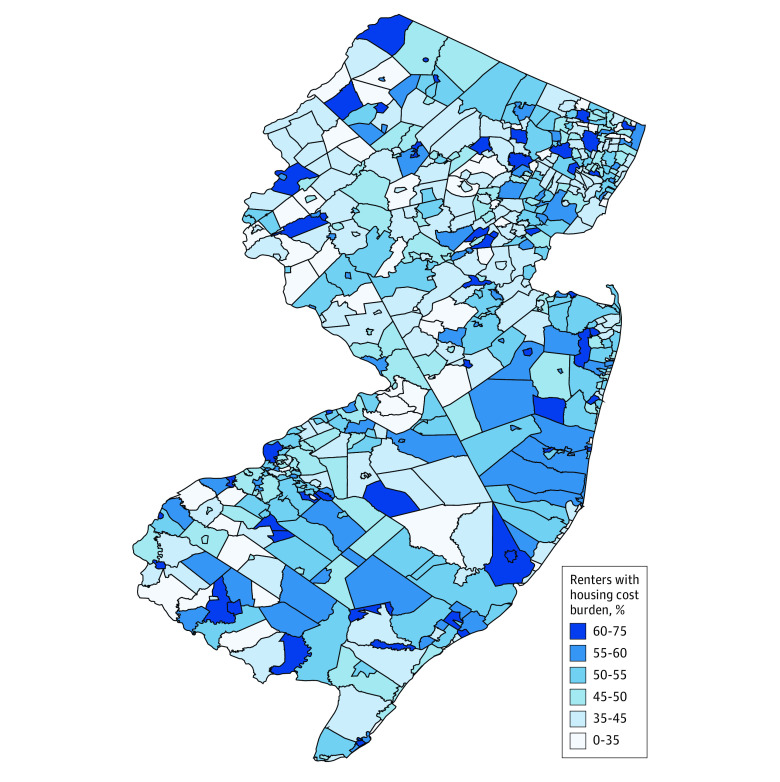
Percentage of Renter Households With Housing Cost Burden in New Jersey Municipalities Housing cost burden was defined as spending more than 30% of household income on rent. Data were obtained from American Community Survey 5-year estimates. Municipalities were self-governing administrative divisions (ie, cities, towns, townships, boroughs, or villages) incorporated under state law.^31^

There was substantial variation in housing cost burden across municipalities (Figure 1). Regardless of which measure of housing cost burden was used (model 1), living in a municipality with a higher rental cost burden was associated with significantly higher odds of SMM (odds ratio [OR], 1.45; 95% CI, 1.17-1.80) (Table 2). Even after controlling for individual- and municipal-level factors (model 2), a 1 percentage point greater rental cost burden (as measured by the percentage of residents spending >30% of income on rent) was associated with 0.27% greater odds of SMM (OR, 1.27; 95% CI, 1.01-1.60). Analyses involving the interaction between housing cost burden and educational attainment (model 3) revealed that the odds of SMM were highest among mothers with less than a high school education (OR, 1.81; 95% CI, 1.06-3.10).

**Table 2.** Association Between Severe Maternal Morbidity and Housing Cost Burden by Indicator of Housing Cost Burden^a^

Indicator	OR (95% CI)			*P* value for interaction
	Model 1	Model 2	Model 3	
**Housing cost burden^b^**				
Percentage with housing cost burden^c^	1.45 (1.17-1.80)	1.27 (1.01-1.60)	NA	NA
Interaction between percentage with housing cost burden and educational attainment				
Educational attainment overall	NA	NA	NA	.12
Less than high school^c^	NA	NA	1.81 (1.06-3.10)	NA^d^
High school^c^	NA	NA	1.11 (0.77-1.59)	.09
Some college^c^	NA	NA	0.99 (0.67-1.45)	.045
College or higher^c^	NA	NA	1.42 (1.06-1.89)	.40
Variance of municipal-level random effect (95% CI)	0.03 (0.02-0.04)	0.03 (0.02-0.04)	0.03 (0.02-0.04)	NA
**Severe housing cost burden** ^e^				
Percentage with severe housing cost burden^c^	1.51 (1.16-1.96)	1.38 (1.04-1.82)	NA	NA
Interaction between severe housing cost burden and educational attainment				
Educational attainment overall	NA	NA	NA	.002
Less than high school^c^	NA	NA	3.56 (1.92-6.62)	NA^d^
High school^c^	NA	NA	1.26 (0.81-1.95)	.002
Some college^c^	NA	NA	0.94 (0.59-1.51)	<.001
College or higher^c^	NA	NA	1.43 (1.01-2.02)	.006
Variance of municipal-level random effect (95% CI)	0.03 (0.02-0.05)	0.03 (0.02-0.04)	0.03 (0.02-0.03)	NA
**Rent to income ratio**				
Median rent to income ratio^c^	1.98 (1.35-2.91)	1.73 (1.15-2.59)	NA	NA
Interaction between median rent to income ratio and educational attainment				
Educational attainment overall	NA	NA	NA	.003
Less than high school^c^	NA	NA	5.11 (2.20-11.90)	NA^d^
High school^c^	NA	NA	1.38 (0.74-2.54)	.004
Some college^c^	NA	NA	0.95 (0.49-1.85)	.001
College or higher^c^	NA	NA	2.02 (1.22-3.35)	.043
Variance of municipal-level random effect (95% CI)	0.03 (0.02-0.04)	0.03 (0.02-0.03)	0.03 (0.02-0.03)	NA

Abbreviations: NA, not applicable; OR, odds ratio.

^a^ All models controlled for individual-level factors (educational attainment, age, race and ethnicity, parity, and year of birth). Models 2 and 3 also controlled for municipal-level factors (median rent, percentage of residents with income lower than the poverty level, and population size).

^b^ Defined as spending more than 30% of income on rent.

^c^ Expressed as a proportion (ie, divided by 100).

^d^ Reference variable.

^e^ Defined as spending more than 50% of income on rent.

**Figure 2.**
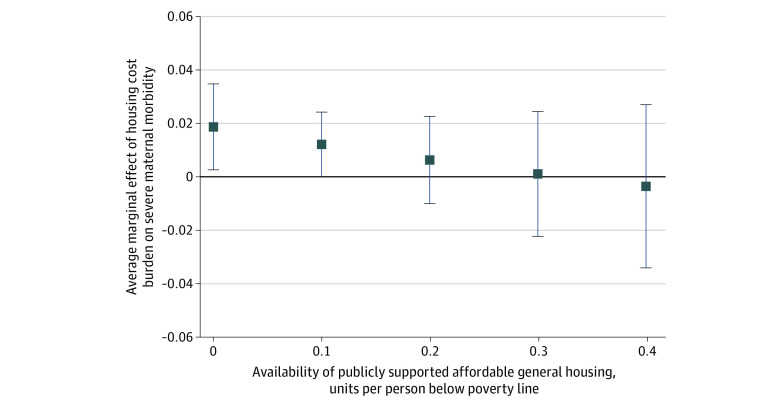
Marginal Effects Associated With Housing Cost Burden in Municipalities by Availability of Publicly Supported Affordable Housing Among Individuals With Less Than High School Education Estimates were derived from multilevel logistic regression models of the association between the percentage of renter households with housing cost burden, the availability of publicly supported affordable housing, and educational attainment with severe maternal morbidity (including analysis of 2-way and 3-way interactions between the 3 exposures). The analysis controlled for individual-level factors (age, race and ethnicity, parity, and year of birth) and municipal-level factors (median rent, percentage of residents with income lower than the poverty level, and population size). Whiskers indicate 95% CIs.

The positive association between rental cost burden and SMM among mothers with lower levels of education decreased at higher levels of affordable housing availability. To ease interpretation, we plotted the average marginal effects associated with rental cost burden, with educational attainment set at less than high school and affordable housing availability levels set at 0, 0.1, 0.2, 0.3, or 0.4 housing units per person with an income lower than the poverty level (Figure 2), representing approximately 0 times, 1 time, 2 times, 3 times, and 4 times, respectively, the mean (SD) municipal-level availability of publicly supported affordable housing of 0.11 (0.20) for the sample (Table 1). In municipalities with no publicly supported affordable housing units, a 1 percentage point greater rental cost burden among renters in the municipality was associated with a 0.019 percentage point (95% CI, 0.003-0.035 percentage points) higher probability of SMM (73.0% of the mean for mothers with less than a high school education) (Figure 2). The probability decreased to 0.012 percentage points (95% CI, 0-0.024 percentage points) in municipalities with 0.2 affordable housing units per person with an income lower than the poverty level, and the probability was not statistically significant in municipalities with 0.3 or 0.4 affordable housing units per person. A sensitivity analysis controlling for hospital of delivery fixed effects yielded similar findings (data not shown).

Among mothers with less than a high school education, the risk of SMM was 8.0% lower for each additional $1000 in annual municipal-level housing subsidy per person with an income lower than the poverty level (risk ratio, 0.92; 95% CI, 0.85-1.00) (eTable 3 in the Supplement). The rates of SMM were 260.4 per 10 000 persons among those with less than a high school education and 159.9 per 10 000 persons among those with a college education, suggesting that an additional $1000 in housing subsidy was associated with a 20.7% (260.4 multiplied by 8% divided by the difference between 260.4 and 159.9) lower overall disparity in SMM between mothers with a college education and those with less than a high school education. Living in a municipality with greater availability of affordable housing designated for older adults did not have a similar moderating effect on the association between housing cost burden and SMM among mothers with less than a high school education (eFigure in the Supplement).

## Discussion

This cross-sectional study found that living in a municipality with higher rental housing costs relative to income was associated with higher odds of SMM. The association was robust to controlling for individual- and municipal-level characteristics and using alternative measures of municipal rental housing cost burden. As hypothesized, the association between rental cost burden and SMM was significant, with the highest odds among mothers with less than a high school education, which is considered a key indicator of low socioeconomic status. We also found that living in a municipality with greater availability of publicly supported affordable housing units attenuated the associations between rental cost burden and SMM for that group.

In the US, affordable housing programs are often controversial, with opponents emphasizing their consequences for property values, property taxes, and crime^27^ and not considering potentially important health impacts. Our finding that publicly supported affordable housing was significantly associated with declines in SMM suggests that the net costs of affordable housing policies to society are lower than typically thought.^34^ Our estimates suggested that an annual $1000 housing subsidy was associated with a substantial (20.7%) reduction in the gap in SMM rates between mothers with a college education and those with less than a high school education. Because SMM confers substantial costs to mothers, children, families, and society,^35^ housing subsidies may have positive implications for other health, economic, and educational outcomes.

Although there is a large body of literature reporting associations between housing and health, relatively little research has focused on the dimension of housing affordability. The few existing studies have relied on survey data and found associations between difficulty with affording the costs of housing and self-reported poor health, hypertension (a factor associated with SMM^36^), and arthritis, but not with heart disease, diabetes, or obesity.^6,7^ Our study contributes to this scant literature by considering a salient health outcome (SMM) constructed from diagnosis codes using CDC criteria and measures of housing cost burden in the municipalities in which individuals reside, which can have consequences for individuals either directly (eg, through stress associated with lack of financial resources or exposure to health-compromising living conditions) or indirectly (through reduced access to health care, nutritious food, or social capital).

A related body of research has found associations between housing instability (primarily homelessness) during pregnancy and adverse maternal and infant health outcomes.^37,38^ Our study contributes to this literature by investigating associations between housing affordability (which can have implications for housing stability) and SMM and adds to mounting evidence that housing characteristics beyond the physical features of buildings can have substantial consequences for health.

Our findings were also consistent with those of a recent study^14^ that found increased levels of municipal spending on housing and community development were associated with lower odds of SMM. Expenditures on housing and community development could include affordable housing but could also encompass redevelopment designed to benefit affluent residents and businesses. In contrast, the official counts of affordable housing units used in this study represented direct measures of subsidized housing available to residents.

### Limitations

This study has several limitations. Although the results were robust to controlling for individual- and municipal-level characteristics, and analyses included a placebo test (defined as a separate analysis of an exposure that should not be effective) in which the availability of affordable housing specifically for older adults (for whom there should have been, and was, no association with SMM) was assessed, the study was not designed to identify causal relationships between SMM, housing costs, and the availability of affordable housing. Our study may have underestimated the number of women with SMM because it did not capture antepartum SMM or SMM that resulted in emergency department or outpatient visits. We assumed that mothers resided in the same municipality throughout the perinatal period, and the results are from a single state and may not be generalizable to other states. Some of the estimates had wide CIs or were significant at the *P* = .05 but not *P* = .01 level.

## Conclusions

The findings of this cross-sectional study suggest that provision of affordable housing may be an actionable strategy for potentially improving maternal health and reducing socioeconomic disparities in maternal health. However, to more fully address the issue of housing cost burden and its consequences for health, it will be necessary to address societal factors that intersect with housing costs, such as redlining (ie, denying financial or other services to individuals wishing to live in a certain area based on their race or ethnicity) and other manifestations of structural racism.

## Notes

Supplement. **eAppendix 1.** Affordable Housing Policy Reform, New Jersey, 1985-2008 **eTable 1.** Affordable Housing Units by Program, New Jersey **eTable 2.** Severe Maternal Morbidity Indicators **eAppendix 2.** Testing for Spatial Autocorrelation **eTable 3.** Association Between Affordable Housing Subsidy in Municipality of Residence and Severe Maternal Morbidity **eFigure.** Marginal Effects (and 95% CIs) of Housing Cost Burden in Municipalities by Availability of Publicly Supported Affordable Housing for Older Adults Among Individuals With Less Than High School Education
eReferences
