# Supplementary material for: Association Between Housing Affordability and Severe Maternal Morbidity
Source: JAMA Netw Open. 2022 Nov 22;5(11):e2243225. doi: 10.1001/jamanetworkopen.2022.43225 (PMC9682423; doi:10.1001/jamanetworkopen.2022.43225)
Supplement: Supplement. — eAppendix 1. Affordable Housing Policy Reform, New Jersey, 1985-2008 eTable 1. Affordable Housing Units by Program, New Jersey eTable 2. Severe Maternal Morbidity Indicators eAppendix 2. Testing for Spatial Autocorrelation eTable 3. Association Between Affordable Housing Subsidy in Municipality of Residence and Severe Maternal Morbidity eFigure. Marginal Effects (and 95% CIs) of Housing Cost Burden in Municipalities by Availability of Publicly Supported Affordable Housing for Older Adults Among Individuals With Less Than High School Education eReferences [file jamanetwopen-e2243225-s001.pdf]

Supplemental Online Content

Muchomba FM, Teitler J, Reichman NE. Association between housing affordability and severe maternal morbidity. *JAMA Netw Open*. 2022;5(11):e2243225. doi:10.1001/jamanetworkopen.2022.43225

eAppendix 1. Affordable Housing Policy Reform, New Jersey, 1985-2008

eTable 1. Affordable Housing Units by Program, New Jersey

eTable 2. Severe Maternal Morbidity Indicators

eAppendix 2. Testing for Spatial Autocorrelation

eTable 3. Association Between Affordable Housing Subsidy in Municipality of Residence and Severe Maternal Morbidity

eFigure. Marginal Effects (and 95% CIs) of Housing Cost Burden in Municipalities by Availability of Publicly Supported Affordable Housing for Older Adults Among Individuals With Less Than High School Education

eReferences

This supplemental material has been provided by the authors to give readers additional information about their work.

© 2022 Muchomba FM et al. *JAMA Network Open*.

eAppendix 1. Affordable Housing Policy Reform, New Jersey, 1985-2008

In a pair of rulings (*Southern Burlington County N.A.A.C.P. v. Mount Laurel Township I and II*), the New Jersey Supreme Court barred municipalities from using exclusionary zoning to block construction of affordable low-and middle-income housing and held that municipalities had a constitutional obligation to use their zoning power in an affirmative way to provide a fair share of affordable housing. In 1985, the New Jersey legislature responded to these rulings by passing the Fair Housing Act that set up a framework for municipalities to meet their constitutional obligations to provide affordable housing for low- and moderate-income families.[1] There was no funding source specified, and implementation of the law varied across municipalities with some communities opposing the law: the law provided for municipalities to be released from up to half of their affordable housing obligation by funding affordable housing in other municipalities; however, some municipalities opted not to support affordable housing development and instead face lawsuits in the courts. Due to the unbalanced and insufficient provision of affordable housing, New Jersey enacted a second major set of affordable housing reforms in 2008.[2] As part of the reforms, municipalities were no longer able to transfer their housing obligations to other municipalities and a statewide 2.5% fee would be assessed on non-residential development to raise funds for the construction or rehabilitation of affordable housing. In addition to state funds, municipalities continue to use federal and local funds as well as municipal inclusionary zoning[3,4] regulations to support affordable housing development.[5,6] As a result, the availability of public-supported housing units per eligible person in the state has varied substantially across municipalities and over time.[6] Further, the state of New Jersey has more jurisdictions with inclusionary housing than any other state,[7] which is ideal for our analysis of the association between publicly supported affordable housing and severe maternal morbidity.

© 2022 Muchomba FM et al. *JAMA Network Open*.

eTable 1. Affordable Housing Units by Program, New Jersey

**Program providing affordable housing**	**# of units**
**Single program**	
NJ Fair Housing Act – Housing to meet a municipality's NJ constitutional obligation. Some developments are federally funded, some are state funded, and some are inclusionary zoning developments.	24,962
Public Housing – A federal housing program	16,354
Section 221 – A federal housing program	5,168
NJ Housing & Mortgage Finance Agency (HMFA) – Various housing supports including construction of affordable housing with state aid raised from bonds and real estate sales.	4,849
Section 8 – A federal housing program	3,159
Low-income housing tax credit (LIHTC) – A federal housing program	2,990
Section 236 – A federal housing program	1,455
NJ Balanced Housing - Supports construction and conversions of new housing units and renovation of existing housing	1,355
Other programs	1,302
**Multi-program**	
HMFA & LIHTC	6,286
HMFA & Section 8	5,325
HMFA & Section 236	3,526
HMFA, LIHTC & Balanced Housing	1,577
Fair Housing Act, HMFA & LIHTC	3,385
Fair Housing Act & Balanced Housing	2,231
Fair Housing Act, LIHTC & Balanced Housing	1,917
Fair Housing Act & LIHTC	1,904
Fair Housing Act & any other program	7,627
Other combination of programs	5,808

Note: Data are for 2015. Housing for seniors, people with disabilities, or other special populations is not included. Other programs include the federal HOME Investment Partnerships, Section 223, and HOPE VI programs, etc.

© 2022 Muchomba FM et al. *JAMA Network Open*.

eTable 2. Severe Maternal Morbidity Indicators

**Diagnostic Codes**
1. Acute myocardial infarction
2. Aneurysm
3. Acute renal failure
4. Adult respiratory distress syndrome
5. Amniotic fluid embolism
6. Cardiac arrest/ventricular fibrillation
7. Disseminated intravascular coagulation
8. Eclampsia
9. Heart failure/arrest during surgery or procedure
10. Puerperal cerebrovascular disorders
11. Pulmonary edema or Acute heart failure
12. Severe anesthesia complications
13. Sepsis
14. Shock
15. Sickle cell disease with crisis
16. Air and thrombotic embolism
**Procedure Codes**
17. Conversion of cardiac rhythm
18. Blood products transfusion
19. Hysterectomy
20. Temporary tracheostomy
21. Ventilation

Source: The Centers for Disease Control and Prevention

© 2022 Muchomba FM et al. *JAMA Network Open*.

eAppendix 2. Testing for Spatial Autocorrelation

We used a choropleth map to visually assess the geographic clustering in housing cost burden in the state. As shown in Figure 1 of the main article, there was substantial variation in housing cost burden across municipalities but it was not clustered in any region of the state, suggesting no need to analyze higher levels of geography such as counties or regions.

We formally tested for the presence of spatial autocorrelation in the residuals in each model by computing global Moran's I. We specified a queen contiguity spatial weights matrix (i.e. municipalities sharing a boundary or corner were considered neighbors). A Moran's I significantly different from 0 indicates spatial dependence in the data that is unaccounted for in the analysis, which could bias the estimates. None of the global Moran's I statistics calculated were statistically significant, indicating that spatial autocorrelation was not a concern.

© 2022 Muchomba FM et al. *JAMA Network Open*.

eTable 3. Association Between Affordable Housing Subsidy in Municipality of Residence and Severe Maternal Morbidity

	OR	(95% CI)		OR	(95% CI)		RR	(95% CI)
Annual housing subsidy per person below poverty (‘000s)	1.00	(0.97-1.02)		0.92*	(0.85-1.00)		0.92*	(0.85-1.00)
Annual housing subsidy per person below poverty (‘000s) × High School				1.11*	(1.02-1.21)		1.11*	(1.02-1.20)
Annual housing subsidy per person below poverty (‘000s) × Some college				1.13**	(1.04-1.23)		1.13**	(1.03-1.23)
Annual housing subsidy per person below poverty (‘000s) × College or higher				1.07	(0.99-1.17)		1.07	(0.99-1.17)
**Random parameter**								
Municipality-level variance	0.03**	(0.02-0.04)		0.03**	(0.02-0.04)		0.03**	(0.02-0.04)

Note: Odds ratios (OR) are from multilevel logistic regression models and risk ratios (RR) are from a multilevel poisson regression model. The models also controlled for individual- and municipal-level factors, i.e., age, educational attainment, race/ethnicity, parity, year of birth, and municipality-level % with housing cost burden, % below poverty level, population size, and an interaction between educational attainment and % with housing cost burden.
* p<0.05, ** p<0.01.

© 2022 Muchomba FM et al. *JAMA Network Open*.

eFigure. Marginal Effects (and 95% CIs) of Housing Cost Burden in Municipalities by Availability of Publicly Supported Affordable Housing for Older Adults Among Individuals With Less Than High School Education

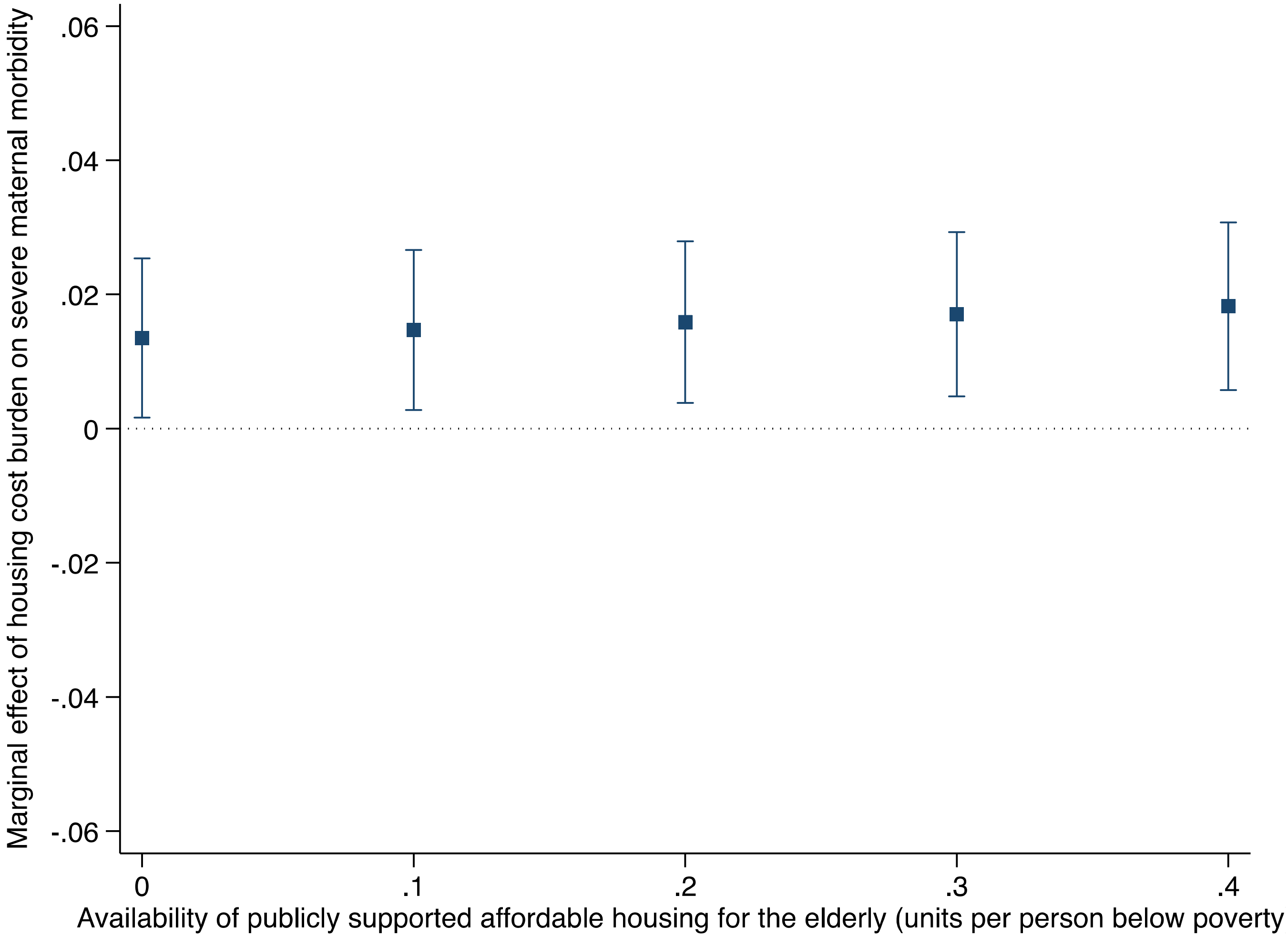

© 2022 Muchomba FM et al. *JAMA Network Open*.

eReferences

1. N.J.A.C. § 5:80-26.1. Housing affordability controls. New Jersey Administrative Code. 2004.

2. Roberts JJ, Jr., Watson Coleman B, Green J, et al. An act concerning affordable housing, revising and supplementing various parts of the statutory law. *New Jersey Legislature.* 2008. https://njleg.state.nj.us/bill-search/2008/A500.

3. Tuller D. Housing and health: The role of inclusionary zoning. *Health Affairs Health Policy Brief*. 2018.

4. Thaden E, Wang R. Inclusionary housing in the United States: Prevalence, impact, and practices. *Lincoln Institute of Land Policy Working Papers.* 2017. https://www.lincolninst.edu/publications/working-papers/inclusionary-housing-in-united-states.

5. Office of Legislative Services. Legislative fiscal estimate. *Office of Legislative Services.* 2008:1-4.

6. New Jersey Department of Community Affairs. Guide to affordable housing in New Jersey. New Jersey Department of Community Affairs Web site. https://www.nj.gov/dca/divisions/codes/publications/guide.html. Updated 2016. Accessed November 26, 2021.

7. Wang R, Balachandran S. Inclusionary housing in the United States: dynamics of local policy and outcomes in diverse markets. *Housing studies.* 2021:1-20. doi:10.1080/02673037.2021.1929863.

© 2022 Muchomba FM et al. *JAMA Network Open*.
